# Revisiting 'Hallmarks of Cancer' In Sarcomas

**DOI:** 10.7150/jca.92844

**Published:** 2024-02-04

**Authors:** Kanya Honoki, Toshifumi Tsujiuchi, Shingo Kishi, Hiroki Kuniyasu

**Affiliations:** 1 Dept. Of Orthopedic Oncology & Reconstructive Medicine, Nara Medical University, Japan.; 2 Dept. of Life Sciences, Kindai University, Japan.; 3 Dept. of Clinical Pathology, Nozaki Tokushukai Hospital, Japan.; 4 Dept. of Molecular Pathology, Nara Medical University, Japan.

**Keywords:** hallmarks of cancer, sarcoma, cancer biology, precision medicine

## Abstract

There is no doubt that anyone who has participated in cancer care or research has once read the 'Hallmarks of Cancer' papers published by Hanahan and Weinberg in 2001 and 2011. They initially defined the six qualities of cancer cells as cancer hallmarks in 2001, but expanded that to 11 as a next generation in 2011.

In their papers, they discussed the potential treatment strategies against cancer corresponding to each of the 11 hallmarks, and to date, proposed therapies that target genes and signaling pathways associated with each of these hallmarks have guided a trail that cancer treatments should take, some of which are now used as standard in clinical practice and some of which have yet to progress that far. Along with the recent advances in cancer research such as genomic analysis with next generation sequencing, they can be reconverged to an alternative six categories defined as selective proliferative advantages, altered stress response, deregulated cellular metabolism, immune modulation and inflammation, tumor microenvironment, tissue invasion and metastasis. In this paper, we will overview the current state of these alternative hallmarks and their corresponding treatments in the current sarcoma practice, then discuss the future direction of sarcoma treatment.

## Introduction

Hanahan and Weinberg published their first influential review paper: the 'Hallmarks of Cancer' 23 years ago to organize the complexities of cancer biology 1, then they published an updating review paper: 'Hallmarks of Cancer: the next generation' a decade later 2, which are comprised with 6 major hallmarks in the first paper and 11 hallmarks in the second paper, i.e. sustained growth signal, evading anti-growth signal, resisting programmed cell death, enabled replicative immortality, inducing new blood flow, tissue invasion and metastasis are the original six, then later added 5 hallmarks: genomic instability, deregulated cellular metabolism, avoiding immune destruction, tumor promoting inflammation, and tumor microenvironment.

The United States National Cancer Act in 1971 have opened the door for the War on Cancer and the clinicians and researchers have been fighting against cancer worldwide. However, Hanahan asked in 2014 whether we are winning the war on cancer over 40 years on, and he concluded that the answer was in general 'No' at that point. He described that despite the introduction of hundreds of new anticancer drugs, including advanced therapies (so-called magic bullets, i.e., molecular targeted agents) aimed at weapons in the enemy's armamentarium, the consensus was that, for most forms of cancer, enduring disease-free responses are rare, and cures even rarer 3. Then, President Obama conducted the NATIONAL CANCER MOONSHOT INITIATIVE in 2016 (www.whitehouse.gov/the-press-office/2016/02/01/fact-sheet-investing-national-cancer-moonshot.).

Under the direction of the Initiative, recent advances of comprehensive genomic analysis such as whole-genome profiling with high through-put next-generation sequencing (NGS) have uncovered numerous aspects of development and progression of cancer. Based on the knowledge from these novel techniques, a lot of molecular targeted therapies have been developed and the strategy for cancer treatments has been proposed as 'precision medicine', a mode of personalized medicine for cancer patients based on the genomic information of each patient. Since then, several onco-panel tests have been applied recently for advanced cancer cases as a companion diagnostic tool for specific agents such as neurotrophic tyrosine receptor kinase (NTRK) inhibitors as well as other molecular targeted therapies. After the years since Hanahan's 'No' critique, there is no doubt that cancer diagnosis and treatments have progressed based on the knowledge of the genomic changes in cancer.

Since the hallmarks proposed by Hanahan and Weinberg are the results of the genetic alterations in cancer cells, their hallmarks have managed the core of cancer biology for understanding the core traits of malignant neoplasms even in this genome-based personalized medicine era. Identifying gene mutations responsible for these hallmarks should establish the molecular mechanism of cancer which could be applied in diagnostic and treatment regimens. However, the cancer biology is far more complicated. Although various drug categories were linked to their targeted hallmarks, numerous implications based on studying these hallmarks have proved to only be effective for a limited time or within limited settings. The reason of this limited clinical efficacy could be that there are so many genetic/epigenetic mutations/alterations as illustrated by the current cancer genome sequencing project, and the cancer genetic landscape is highly diverse without one-to-one relationship between gene mutations and hallmarks.

Sarcomas are not exceptional on this issue. Most sarcomas are lacking the targetable genetic alterations showing low tumor mutational burden and high copy number alterations 4. Boddu et al reported that the majority of alteration were structural (60.5%), most commonly amplification and copy-number loss, and the majority of patients (84.9%) showed low tumor mutational burden (< 6 mutations/Mb) and no patients had evidence of microsatellite instability 5. Although incidence of the patients with sarcoma who have a potentially targetable mutation reach 40-50 % using NGS analysis 5-7, only around 15% patients were influenced by NGS findings on their therapeutic selection 5,7. As a matter of fact, pazopanib, a multi-tyrosine kinase inhibitor, is the only approved molecular targeting agent against advanced soft tissue sarcomas and we have nothing for bone sarcomas at this moment. Although NTRK inhibitors have been approved recently for several cancers including sarcomas with NTRK fusion genes, eribulin and trabectedin, both recently approved for advanced soft tissue sarcomas are not the molecular targeting agents but rather the classical drugs of natural product origins.

**Figure 1:**
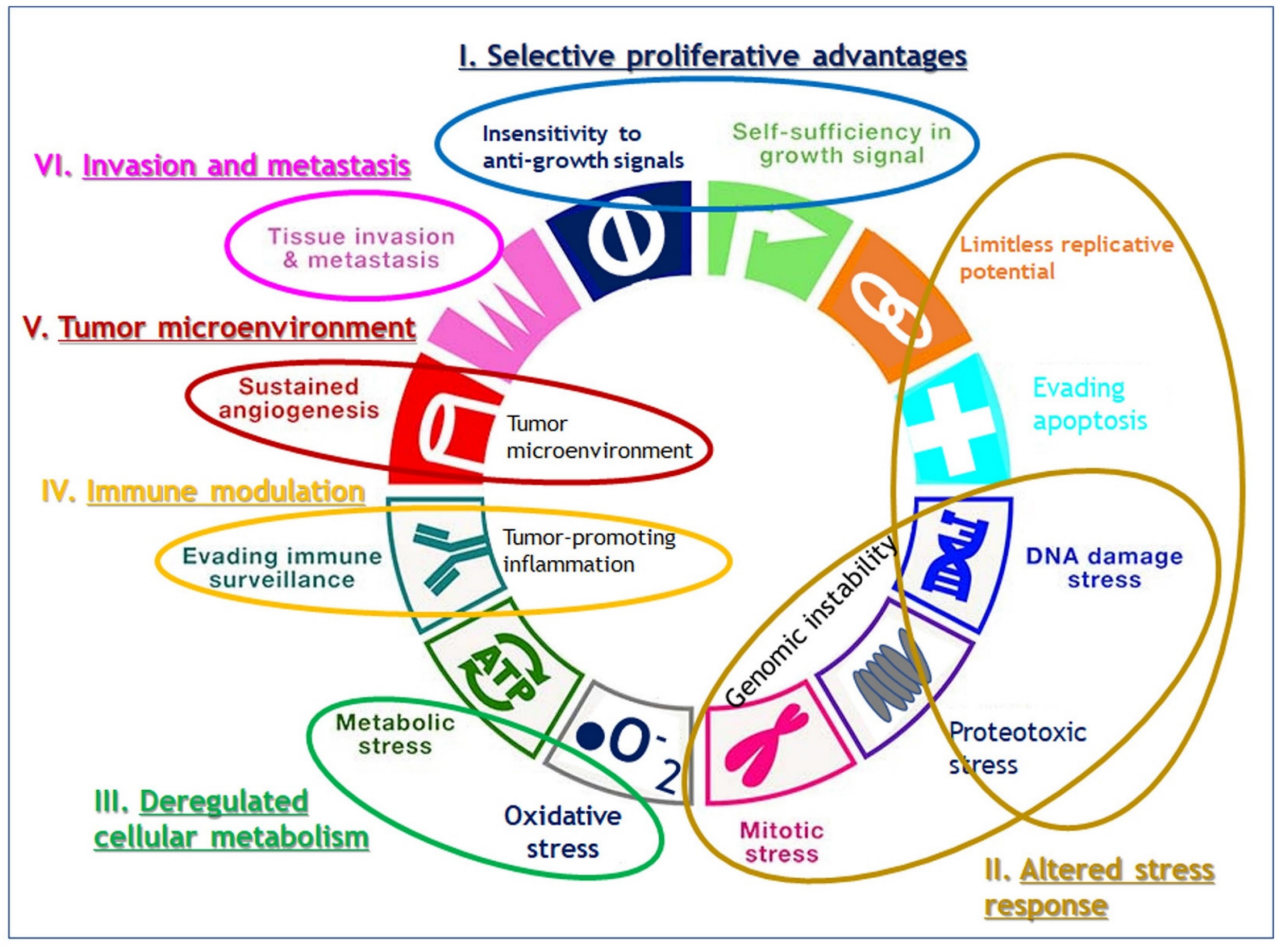
Reorganization of 11 hallmarks into 6 hallmarks by combining the hallmarks which share their roles in cancer evolution and progression. Sustained growth signal and evading anti-growth signal are combined into selective proliferative advantages; resisting programmed cell death and enabled replicative immortality and genome instability into altered stress response; tumor promoting inflammation and avoiding immune destruction into immune modulation, and tumor microenvironment including vascular structure reorganization such as angiogenesis, and the original hallmarks of deregulated cellular metabolism and tissue invasion and metastasis are maintained as they are.

While Weinberg himself admitted that their framework concept of hallmarks came full circle to confronting the endless complexity of cancer 8, their review has still served as blueprints for understanding the core traits of cancer. Since viewing the hallmarks as individual, segregated and static targets is inappropriate, we attempted here to reorganize 11 hallmarks into alternative 6 hallmarks by combining the 2011's hallmarks which share their roles in cancer evolution and progression such as sustained growth signal and evading anti-growth signal into selective proliferative advantages; resisting programmed cell death and enabled replicative immortality and genome instability into altered stress response; tumor promoting inflammation and avoiding immune destruction into immune modulation, and tumor microenvironment including vascular structure reorganization such as angiogenesis (Fig. 1). We maintained the rest of original hallmarks, i.e., deregulated cellular metabolism and tissue invasion and metastasis in the original review.

Here, we reviewed the articles using the keywords of 'hallmarks of cancer' and related to each original 11 hallmarks in sarcomas, and selected the topics with clinical relevance and basic research investigation which supports biological mechanisms including approval in current practice as well as on-going clinical trials as possible. We will overview the current status of the hallmarks and their clinical relevance in sarcomas, and up-to-date an organized picture of hallmarks to seek the future direction of the treatments against sarcomas.

## I. Selective Proliferative Advantages

**Figure 2:**
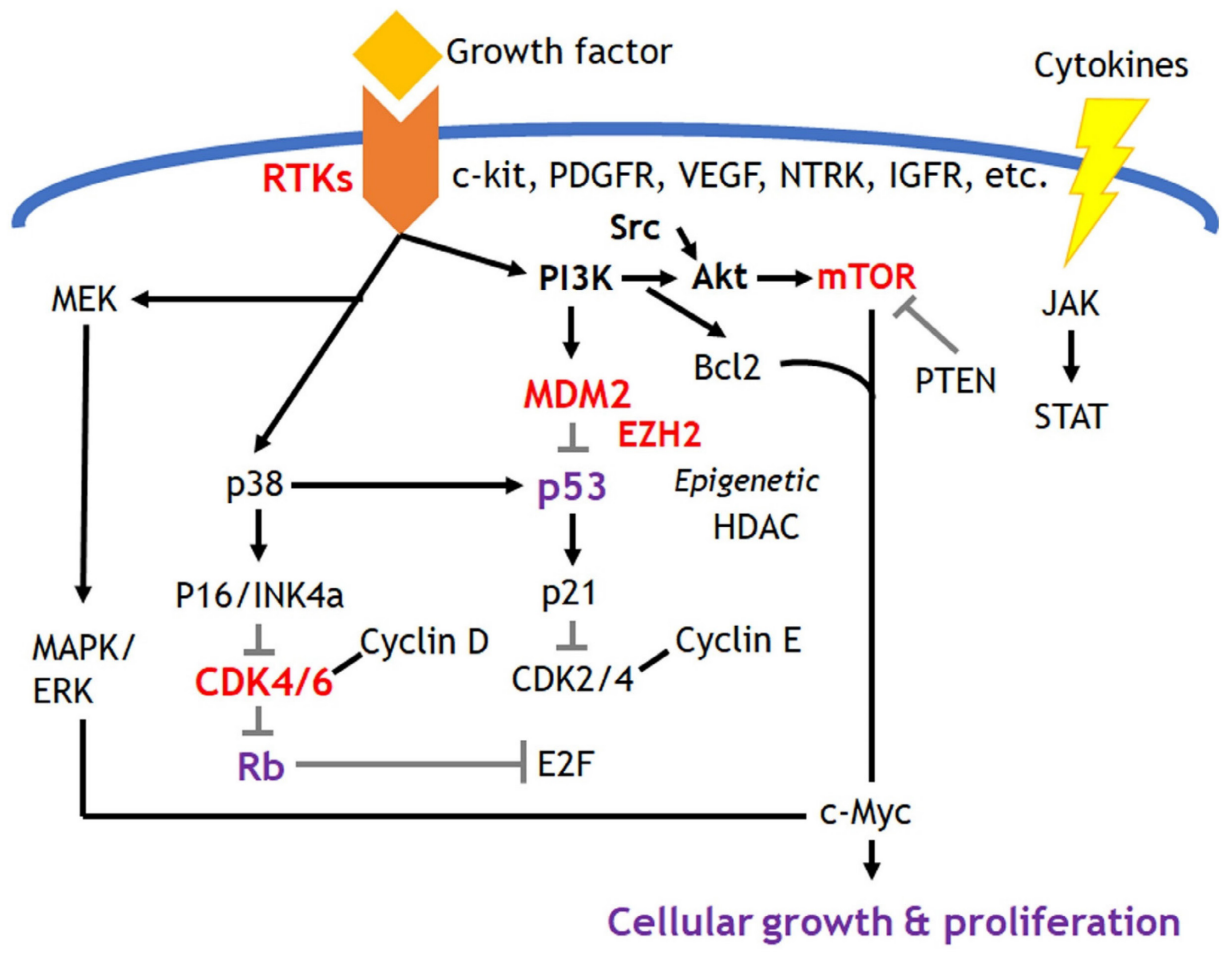
Targeted signaling pathways in sarcomas. Most common targets take a place in receptor tyrosine kinases signaling and cell cycle regulators related with p53 and Rb pathways. Epigenetic regulators such as EZH2 could be the potential therapeutic targets in specific sarcomas.

This hallmark includes sustained growth signal and evading anti-growth signal which are mostly related to the activated growth factor signaling resulted from the gain of oncogenes and the loss of tumor suppressor genes. The development of molecular targeted therapies has been evolving in this field, and Figure 2 depicts the possible targetable pathways. The breakthrough of the most influential targeted treatments among them would be tyrosine kinase inhibitors for the treatment of sarcomas. Factors of platelet-derived growth factor receptor (PDGFR), vascular endothelial growth factor receptor (VEGFR) and c-kit receptor tyrosine kinase receptors are currently targeted in approved therapies. Investigations onto possible therapies targeting insulin-like growth factor 1 receptors (IGF1R), Met receptors and Src tyrosine kinases are also underway in several sarcomas. Receptor tyrosine kinases initiate multiple downstream signaling pathways, such as mitogen-activated protein kinase (MAPK), phosphatidylinositol 3-kinase (PI3K), Janus kinase/signal transducer and transcriptional activator (JAK/STAT), which can initiate multiple downstream signaling pathways and induce various carcinogenic cellular processes such as cell survival, proliferation, differentiation, and evading apoptosis. Imatinib, sunitinib, and pazopanib are inhibitors of these receptor tyrosine kinases and are approved as standard of care options in many countries. Imatinib initially targeted the fusion protein bcr-abl for the treatment of myeloid leukemia. However, it was later found to have an inhibitory effect on c-KIT and PDGFR. Imatinib had a high response rate of 50-70% to the treatment of gastrointestinal stromal tumors (GIST), which have constitutive activation of c-KIT and PDGFR 9, 10. Based on this success in GIST and other preclinical data, attempts have been made to extend its therapeutic application to other sarcoma subtypes with abnormal expression of c-KIT or PDGFR 11, 12. However, most such attempts, including a phase II clinical trial conducted by the Pediatric Oncology Group (COG) to test the efficacy of imatinib in a variety of pediatric sarcomas with high c-KIT or PDGFR levels, have failed with poor response 13-15. COG clinical trials revealed that only 2% of patients had a very partial response to imatinib, concluding that the receptor tyrosine kinase targeted by imatinib is not a molecular driver or a possible alternative pathway to avoid imatinib-induced cell death present in pediatric sarcomas 13, 14.

Another multi-targeted tyrosine kinase inhibitor approved for the treatment against sarcomas is pazopanib which also targets multiple tyrosine kinase proteins such as PDGFR and VEGFR 16, 17. Pazopanib has been approved for the treatment of advanced soft tissue sarcomas in patients who received prior chemotherapy based on the results of a randomized, double-blind, placebo-controlled phase III trial (PALETTE study conducted by van der Graaf WTA, Blay J-Y, Chawla SP, et al) that demonstrated improved median progression-free survival for pazopanib compared to placebo (hazard ratio [HR] 0.31, 95% CI 0.24-0.40; p<0.0001), even though overall survival was not significantly improved 18. Now pazopanib has been widely used for the treatment of advanced soft tissue sarcomas and the success and approval of this drug for soft tissue sarcomas opens the door to expand the indications on metastatic bone sarcomas and the results show that the median overall survival was 11 months and progression free survival was 5.4 months, and 68% of the patients had either partial response or stable disease 19. In addition, another attempt of the use of pazopanib has been conducted as a combinatory treatment with chemotherapy or radiotherapy. The results of phase 2 randomized clinical trial (PAPAGEMO) have been released and indicated that the addition of gemcitabine to pazopanib was tolerable and progression free survival ratio at 12 weeks was significantly higher compared with pazopanib alone 20. These results suggest clinical activity of the combination of pazopanib with gemcitabine against soft tissue sarcomas, and the confirmation with a phase 3 trial will be awaited especially in more homogeneous population such as leiomyosarcoma cases.

The IGF and IGF1R signaling pathway promotes cell survival and proliferation through activation of the PI3K/AKT/mTOR axis 21. Elevated levels of IGF1R and its ligands have been observed in various sarcomas and it correlates with poor prognosis 22. Among various sarcomas, the tumorigenic role of IGF-IGF1R axis has been established in Ewing sarcoma because the IGF1R is a direct target of the EWS-FLI1 fusion oncoprotein 23. Early phase clinical trials encouraged the benefit of IGF1R inhibition against sarcomas, however, as predicted, resistance to the IGF1R inhibitors developed in most cases which initially responded to the therapy, and eventually suffered from relapses 24. Thus, the investigation on the mechanism of resistance and combinatory or additive treatments are underway.

The activation of MET and Src signaling pathways have also been implicated in tumor progression of various sarcomas. Both MET and Src have been suggested their involvement in invasion and metastasis process in sarcomas 25, 26, and MET inhibitor, crizotinib, and Src inhibitor, dasatinib showed inhibitory effects for tumor growth, invasion, and metastasis in bone sarcomas both *in vitro* and *in vivo* 27, 28. The phase II clinical trials conducted by European Organization for Research and Treatment of Cancer (ClinicalTrials.gov identifier: NCT01524926) indicated that crizotinib against MET+ clear cell sarcoma showed comparable results with the results achieved by first-line single-agent doxorubicin in non-selected metastatic soft tissue sarcomas, as well as the results achieved by pazopanib in previously treated sarcoma patients in terms of progression-free survival 29. While, the same trial demonstrated that crizotinib has activity in TFE3-rearranged, MET+ ASPS patients 30. The phase II clinical trials (ClinicalTrials.gov identifier: NCT00788125) are now ongoing to further evaluate the use of dasatinib in combination with chemotherapeutic agents including ifosfamide, carboplatin and etoposide. In addition, another study also suggested that Src family kinase (SFK) inhibition through recently developed selective SFK inhibitor (a pyrazolo[3,4-d] pyrimidine derivative, called SI221) showed reduction of Ewing sarcoma cell viability at least in part by hindering an SFK-NOTCH1 receptor-p38 mitogen-activated protein kinase (MAPK) axis 31.

Mammalian target of rapamycin (mTOR) which is activated in PI3K/AKT pathway has also been considered as the potential target for sarcoma treatment, but early phase of clinical trials showed the disappointing results 15.

Deregulation of the CDKN2A-CCND-CDK4/6-retinoblastoma 1 (Rb) pathway is frequently observed in about 25% of unselected sarcomas and is a distinct pathogenic characteristic for specific subtypes. Recently, two comprehensive genetic analysis of thousands of sarcomas with more than 40 pathological entities have been reported using MSK-IMPACT and Foundation One HEME^TM^ 32,33. Both reports described that most common alterations were in cell cycle control, p53 and Rb1, receptor tyrosine kinase/PI3K in addition to the low frequencies of both tumor mutational burden and microsatellite instability, and several subtype-specific associations such as SAMRCB1 deletion in epithelioid sarcoma and malignant rhabdoid tumor as well as cyclin-dependent kinase 4 (CDK4) and mouse double minute 2 homolog (MDM2) amplification in well/dedifferentiated liposarcoma. These genomic specificities have fueled the clinical evaluation of selective CDK4/6 inhibitors in sarcomas. CDKs are accelerator of cell cycle with binding the cyclins. For instance, CDK1 binds to cyclin A/B to facilitate G2 to M transition and CDK4 binds with cyclin D and the complexes phosphorylate the RB protein to release the E2F which activates CDK2/cyclin E complexes for facilitating G1 to S phase progression. Classically, flavopiridol, a potent multiple CDKs (2,4,6 and 7) inhibitor has been shown its efficacy against various types of cancers including sarcoma 34-36 but a phase II study in patients with previously untreated advanced soft tissue sarcoma failed to show its efficacy and concluded that the use of flavopiridol had no objective treatment responses and could not support further exploration of flavopiridol as a monotherapy in soft tissue sarcomas 37. CDK1 inhibitors currently are not available for sarcomas, as well as CDK4/6 targeted inhibitors such as palvociclib and ribociclib which are approved for breast cancer by the US Food and Drug Administration (FDA) also have not been approved yet for sarcomas. However, it could be possible that they will have a potential to show the efficacy against sarcomas primarily driven by CDK4/6 deregulation such as dedifferentiated liposarcomas. In addition, in subtypes with mTOR overexpression or PTEN loss with alteration in the Rb-CDK4/6 pathway as a secondary driver such as leiomyosarcoma, angiosarcoma and osteosarcoma, combination therapies with CDK inhibitors and mTOR or PI3K inhibitors might be a potential therapeutic option. In this context, alterations of CDK4, CCND, CCNE, RB1, E2F1, and CDKN2A have been proposed as potential biomarkers for CDK4/6 inhibitor in sarcoma 38. Although the use of CDK4/6 inhibitors across sarcomas remains limited, specific subtypes could be sensitive to this therapeutic strategy as either monotherapy or combination therapy. In addition to CDK inhibitors, MDM2 inhibitors such as milademetan are the possible considerable candidates for the targeted treatments of certain types of sarcomas including liposarcomas, and clinical trials of milademetan has been on-going, showing that the disease control rate and median progression-free survival were 58.5% (95% CI, 44.1 to 71.9) and 7.2 months overall (n=53), and 62.0% (95% CI, 35.4 to 84.8) and 7.4 months against dedifferentiated liposarcomas with the recommended intermittent schedule (n=16), respectively 39. This notable effect of milademetan in dedifferentiated liposarcomas has evoked randomized phase III trial (MANTRA).

Another possible target could be epigenetic regulators such as histone deacetylase (HDAC) inhibitors and EZH2 inhibitor. SMARCB1 (BAF47/INI1) deletion has been found in several malignant tumors including epithelioid sarcoma. SMARCB1 is a subunit of the SWI/SNF chromatin remodeling complex that opposes the enzymatic function of EZH2. When INI1 loses its regulatory function, EZH2 activity is up-regulated, allowing EZH2 to play a driving force of cancer development 40. Tazemetostat, a specific EZH2 inhibitor, has just been approved for patients with advanced epithelioid sarcoma and represents a new therapeutic option for this disease 41.

Future perspectives: To advance the optimal clinical usage of molecular targeted therapeutics including the combined treatments, identification of the molecular markers for each drug will be required to promote the so-called precision medicine.

## II. Altered Stress Response

Cells adopt a variety of responses to adapt to the stress, and the stress response mechanisms are often subverted or hijacked for overall cell survival on the road into cancer cells. These stress responses include DNA repair, apoptosis, autophagy, senescence, and metabolic rewriting, those of which involve the signaling pathways such as ataxia-telangiectasia mutated (ATM)/ ATM-and Rad3-related (ATR), checkpoint kinase (Chk)1/2, and AMP-activated protein kinase (AMPK).

Tomasetti and Vogelstein discussed an interesting correlation (r=0.804) between estimated lifetime stem cell division number in 31 tissue types and corresponding cancer incidence rates in the United States 42. They categorized two entities in various tumors, R-tumor (replicative) showing low extra risk and D-tumor (deterministic) showing high extra risk, and concluded that the majority of cancers including osteosarcomas is categorized as R-tumor due to “bad luck,” that is, random mutations arising during DNA replication in noncancerous normal stem cells. In colorectal cancer, Vogelstein's initiation-progression model has been widely accepted as the carcinogenesis model 43. The model is known as 'adenoma-carcinoma sequence' which proposed the stepwise accumulation of genetic changes on certain genes such as APC, k-Ras and p53 could contribute to the development of familial adenomatous polyposis (FAP)-related and sporadic colorectal cancer. On the other hand, colorectal cancer that occurs in patients with hereditary non-polyposis colorectal cancer (HNPCC) involves mutations in mismatch repair gene such as hMLH1 and hMSH2 and microsatellite instability (MSI), suggesting that there is not one carcinogenic route for cancer development.

Whole genome or whole exome analysis with next generation sequencing revealed several aspects of oncogenesis in variety of cancers. In case of transformation of dedifferentiated liposarcomas from atypical lipomatous tumors, so-called well differentiated liposarcomas, accumulation of genetic alterations including loss of function of p53, somatic copy number alterations and other genetic changes could contribute to the malignant transformation 44. However, the stepwise accumulation of genetic alterations might not be adapted to the most sarcomagenesis and it seems this phenomenon occurs in rare occasion in sarcomagenesis. As has been previously reported, most sarcomas are considered to have few somatic mutations.

**Figure 3:**
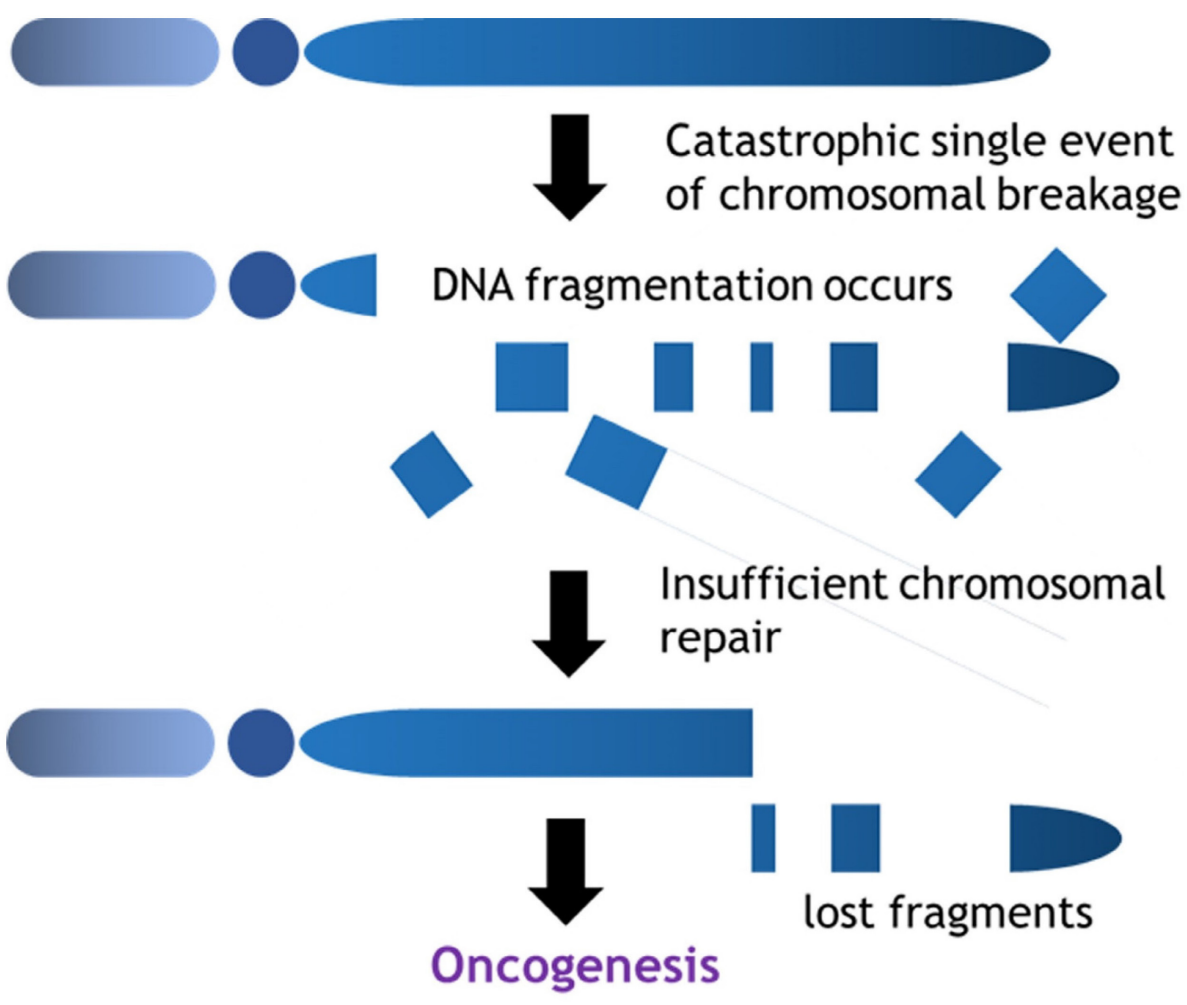
Chromothripsis is a catastrophic single event which extensively results in DNA fragmentation. Certain portions of chromosomes are lost to the cells during the chromosomal repair and these mis-rearrangements progress towards cancer formation.

In contrast, the prevalence of chromothripsis is relatively high in sarcomas amongst various malignant tumors 45. Chromothripsis is characterized by massive genomic rearrangements localized to isolated chromosomal regions and is often generated by a single catastrophic event (Fig. 3). In contrast to the process of the accumulation of mutations as mentioned above, chromothripsis provides a mechanism for the rapid accrual of hundreds of rearrangements in a few cell divisions. Chromothripsis links to entity-specific drivers (oncogene activation, fusion gene formation etc.), telomere attrition (TERT gain, ATRX truncation (alternative lengthening of telomeres)), mutational signature, clonal heterogeneity. Voronina et al reported the prevalence of chromothripsis in various cancer types, indicating the higher prevalence in sarcomas; malignant peripheral nerve sheath tumor: 100%; leiomyosarcoma: 78%; osteosarcoma: 74%; liposarcoma: 60%; sarcoma, NOS: 55% 46. Whole genome or whole exome analysis in osteosarcomas suggested that high confidence of chromothripsis score reached 74% affecting multiple chromosomes with highly involvement of telomere (79%) and centromere (86%) region. Chromothripsis would be the fundamental cause of tumor heterogeneity and theoretically could occur during the entire process of tumor evolution from tumor initiation through the development of metastatic foci. In addition, the different pattern of chromothripsis might be observed in the different portion of the bulk of a tumor in terms of genetic heterogeneity.

**Figure 4:**
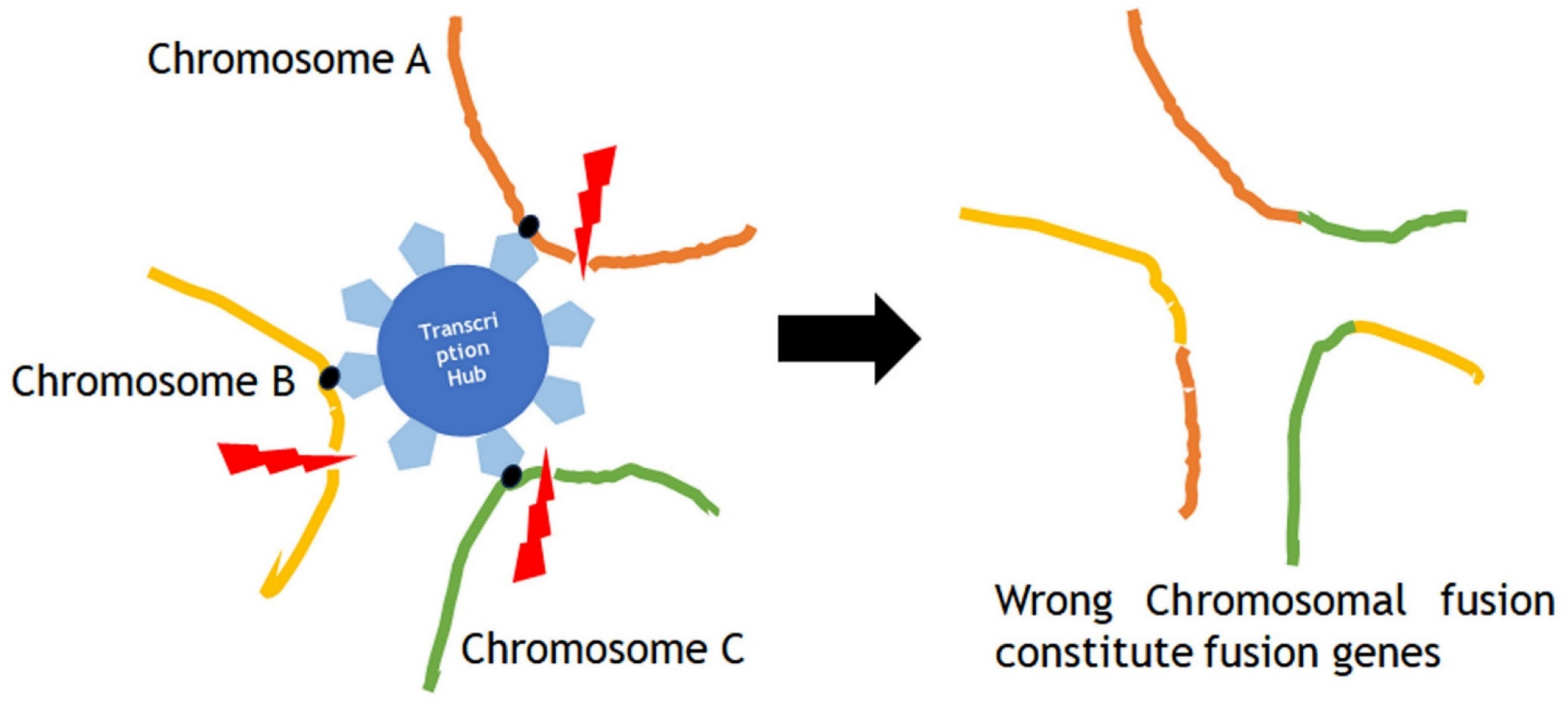
Chromoplexy occurs in a form of ruptures in multiple localized chromosomes at the same time in the nuclear transcription hub, which simultaneously regulates the expression of multiple genes on multiple chromosomes in close proximity, and in the process of repair, different chromosomes mistakenly fuse with each other, which is involved in carcinogenesis.

Chromoplexy and kataegis are also involved in carcinogenesis. Sarcomas are often defined by characteristic gene fusions which are the results of chromosomal structure rearrangement, for instance the EWSR1-ETS fusions in Ewing sarcoma. The common mechanism of fusion gene generation has been considered as a simple reciprocal translocation. Anderson et al investigated the genesis of EWSR1-ETS fusions through whole-exome or whole-genome sequencing data from 124 patients with Ewing sarcoma, and their analysis of structural rearrangements revealed that in 52 of 124 (42%) tumors, the EWSR1-ETS fusion arose by chromoplexy, a sudden burst of complex, loop-like rearrangements 47. Chromoplexy occurs in a form of ruptures in multiple localized chromosomes at the same time in the nuclear transcription hub, which simultaneously regulates the expression of multiple genes on multiple chromosomes in close proximity, and in the process of repair, different chromosomes mistakenly fuse with each other, which is involved in carcinogenesis (Fig. 4). Recurrent chromoplexy-generated fusions are not limited to Ewing sarcoma, but detected in chondromyxoid fibroma, synovial sarcoma and phosphaturic mesenchymal tumor, suggesting that chromoplexy is a novel molecular mechanism widely involved in the development of sarcomas 47.

Chk1 inhibitors has been developed and demonstrated for instance that inhibition of Chk1 sensitized the Ewing sarcoma cells to gemcitabine 48, however has not been clinically approved against sarcomas at this moment.

Besides targeting the genomic alteration, epigenetic regulators such as DNA methylation, histone modification and microRNA are emerging potential targets against sarcomas including tazemetostat, an EZH2 inhibitor for epithelioid sarcoma with loss of SMARCB1/INI1/BAF47 as mentioned above.

Future perspective: Mechanisms of genomic rearrangements might be difficult as the direct therapeutic targets, and most sarcomas lack the targetable specific genetic mutations. Epigenetic regulators could be more feasible and realistic targets in this hallmark of sarcomas.

## III. Deregulated Cellular Metabolism

**Figure 5:**
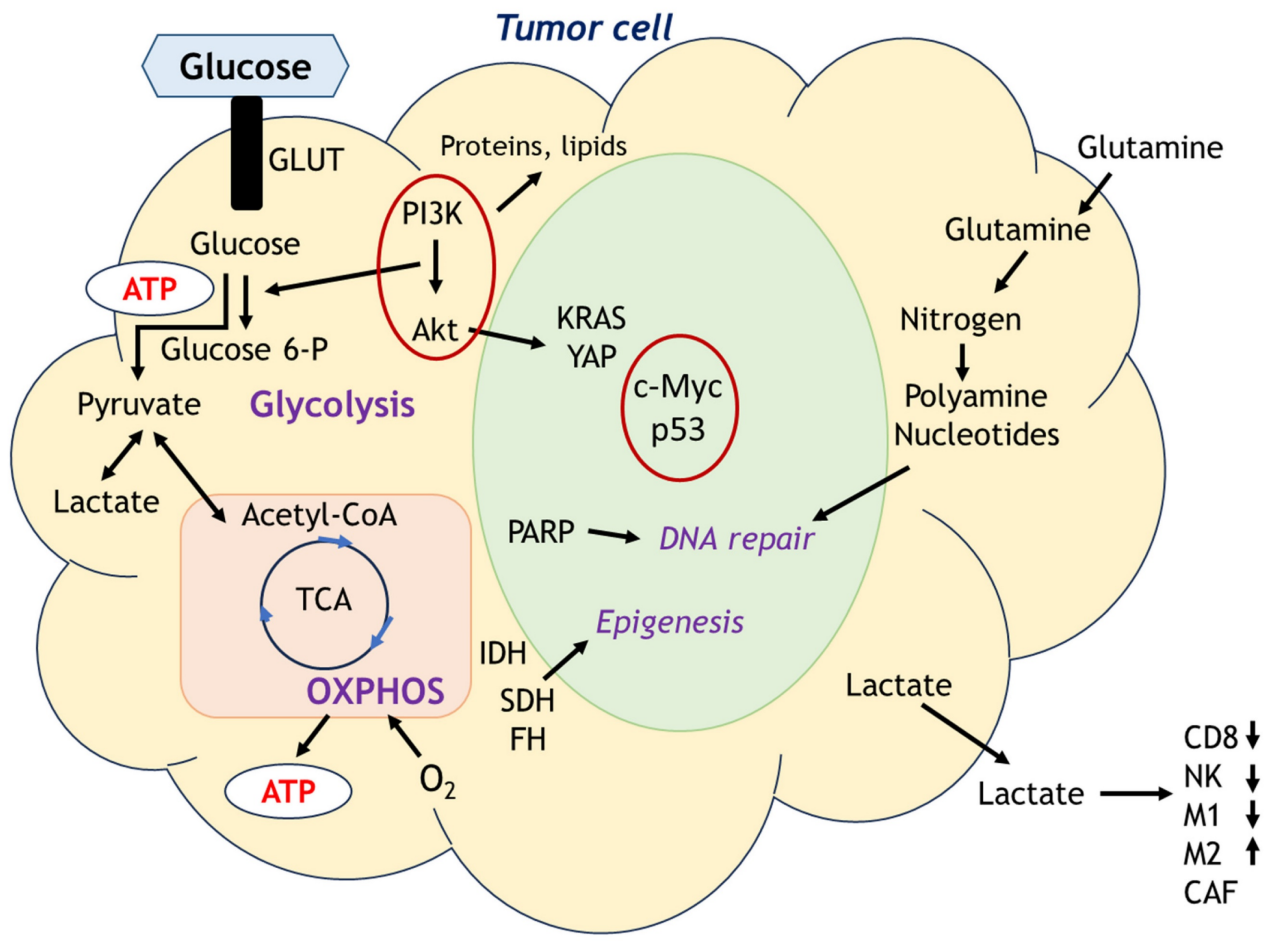
Main sources of cell energy are glycolysis and OXPHOS, but various metabolites involve the many aspects of cellular activities such as nucleotides generation, immune cell function.

In 1930, Otto Warburg, a pioneer in cancer metabolism research, described that cancer cell energy metabolism heavily shifted to aerobic glycolysis even in oxygen-rich environments, known as Warburg effect after his name 49. Since then, the metabolic adaptations of cancer cells have been extensively investigated as metabolic rewiring or metabolic reprogramming which is defined as the increase or suppression of standard metabolic pathways activity through tumorigenic mutations and providing the power to fulfill their biosynthetic, bioenergetic and redox balance needs in cancer cells 50. Esperança-Martins et al. comprehensively reviewed sarcoma metabolomics based on the summary by Pavlova and Thompson 51,52. They have been reshaping the six metabolic hallmarks focusing on changes in metabolic processes that supply the energy required for cancer cell proliferation. Figure 5 depicts the metabolic cascade in tumor cells. Main sources of cellular energy are glycolysis and OXPHOS, but various metabolites involve the many aspects of cellular activities such as nucleotides generation, immune cell function. The metabolism of cancer cells is triggered by mutations in numerous genes, such as c-Myc, k-RAS, c-Src, YAP, PI3K/Akt, loss-of-function mutation in tumor suppressor p53, and elevated HIF levels 53,54. These mutations can stimulate glucose uptake and glycolysis. Other metabolic processes, such as IDH gain-of-function mutations and metabolic plasticity relative to local oxygen availability, can also affect cancer cell proliferation 55,56. Mutations in cancer cell metabolism genes are also involved in the production of chromatin-modifying metabolites, including loss-of-function succinate dehydrogenase (SDH) and fumarate hydratase (FH) mutations, as well as IDH mutations, which can lead to the accumulation of 2-hydroxyglutarate and downregulation of tumor suppressor genes and cellular differentiation blockade via DNA and histone hypermethylation 55.

Depend on the condition of cell states, central molecules in cell metabolism would be different. In proliferative state cells, glucose is metabolized mainly through pentose phosphate pathway (PPP) and ATP has been produced from TCA cycle via respiratory electron-transfer chain intervened by acetyl-CoA coming either from pyruvate, fatty acids and amino acids oxidation. In hypoxic condition, PPP is inhibited and glucose is metabolized through anaerobic glycolytic pathway. In a low glucose condition, glutaminolysis and fatty acids oxidation could be a main source of energy production. Inhibitors of glucose metabolism such as 2-deoxy-D-glucose (2-DG), a competitive inhibitor of glycolysis, effectively reduced the viability of sarcoma cells such as alveolar rhabdomyosarcoma 57, however the clinical application of 2-DG has not been achieved yet against sarcomas.

Cancer cell subpopulations are heterogenous regarding nutrient requirements and metabolic adaptations to accomplish biosynthetic and bioenergetic purposes. So-called cancer stem cells (CSCs) are supposed to consist of a tiny subpopulation of cancer cells within heterogeneous tumor cells that are undifferentiated, with self-renewal capability. They can initiate, propagate, and spread the cancer, being as a precious reservoir of potential distinct differentiated tumor cells, and contribute to drug resistance and tumor relapse. CSCs from different tumors show specific energetic and metabolic pathways, even though oxidative phosphorylation (OXPHOS) and glycolysis remain the primary energy production mechanisms 58. Palorini et al have shown that osteosarcoma CSCs 3AB-OS cells are more dependent on high glycolysis and less dependent on OXPHOS for energy production and survival when compared with OS MG63 cells (non-CSCs) 59. Controversially, CSCs are supposed to be quiescent tumor cell subpopulations, and they could tend to significantly less glycolytic and show higher dependence on OXPHOS with elevated expression of mitochondrial respiratory components and using carbon predominantly for bioenergetic purposes 52. Pterostilbene (PTE), a plant's polyphenol, showed the inhibitory effect of osteosarcoma (OS) cell growth and sphere forming ability and stem cell marker expressions. The mechanism of the effect of PTE on OS cells is promoting mitochondrial ROS generation and membrane depolarization through the suppression of OXPHOS, reducing oxygen consumption rate and ATP synthesis via F0F1-ATP synthase inhibition. PTE showed the enhanced anti-cancer efficacy with c-Myc inhibitors, proposing dual inhibition of OXPHOS and glycolysis might be a potential therapeutic candidate against osteosarcoma 60.

Reshaped metabolome in sarcomas by deregulated pathways could be potential therapeutic targets, showing the efficacy by other targeted therapies through direct or indirect modulation of the metabolome. For example, PI3K/Akt/mTOR pathway directly controls protein and lipid synthesis and glucose metabolism 61-66. Another possible metabolic target in cancer cells is poly (ADP-ribose) polymerases (PARP) which is related to repairing damaged or abnormal DNA represented by activated PARP-1 67,68. PARP inhibitors (PARPi) have represented a potential lethal approach against various cancer cells with specific DNA-repair defects. In Ewing sarcomas, both catalytic PARP inhibition and PARP-DNA trapping showed the antitumor activity in preclinical and clinical studies 69. Although studies in preclinical *in vivo* models and clinical trials of PARPis have disappointingly failed to demonstrate worthwhile response in Ewing sarcoma patients 70, the synergistic efficacy combining PARPis with nicotinamide phosphoribosyl transferase inhibitors (NAMPTis) has shown *in vitro* and *in vivo* models in Ewing sarcoma, hopefully showing its potential for use in Ewing sarcoma patients 71.

Metabolomics is an emerging aspect of cancer biology and sarcoma metabolomics is not an exception. Although sarcoma metabolomics is a broadly unexplored field, there is no doubt that deeper characterization and a sharper picture of sarcoma metabolic landscape could provide the potential molecular biomarkers both in diagnostic and therapeutic targets.

Future perspective: Targeting tumor metabolic pathways are still in challenging. Recently, mitochondria transfer from the platelets affects the cancer cell metabolic status modulating oxidative stress which reprogram metastatic state of cancer cells 72. In this hallmark, mitochondria would be a potential candidate for future therapeutic targets.

## IV. Immune Modulation and Inflammation

The role of immune system in cancer has been recognized for decades, and several anticancer immunotherapies have been developed. Dunn et al demonstrated that the dual function of immunity indicating immune system as both killers of cancer cells and boosters of cancer outgrowth. They hypothesized the cancer immunoediting consisted of three phases, i.e., controlling cancer (elimination, equilibrium) and promoting cancer (escape) 73. The first phase of cancer immunoediting is that the innate and adaptive immune system act in concert to destroy the nascent tumor, thus leading to tumor “elimination”, which is the main tenet of the cancer immunosurveillance hypothesis. The second phase is the cancer outgrowth immunologically restrained, but not eliminated entering the 'equilibrium' 74. Further immunological casting of the tumor and establishment of a suppressive tumor microenvironment (TME) may lead to the 'escape' phase of cancer immunoediting, which manifests the clinically apparent disease of cancer. In this 'escape' phase, cancer immunoediting modifies tumor immunogenicity through neoantigen loss by immunoselection and hijacking pathways which limits inflammatory responses, integrating the hallmarks of 'avoiding immune destruction' and 'tumor-promoting inflammation' 75, 76.

**Figure 6:**
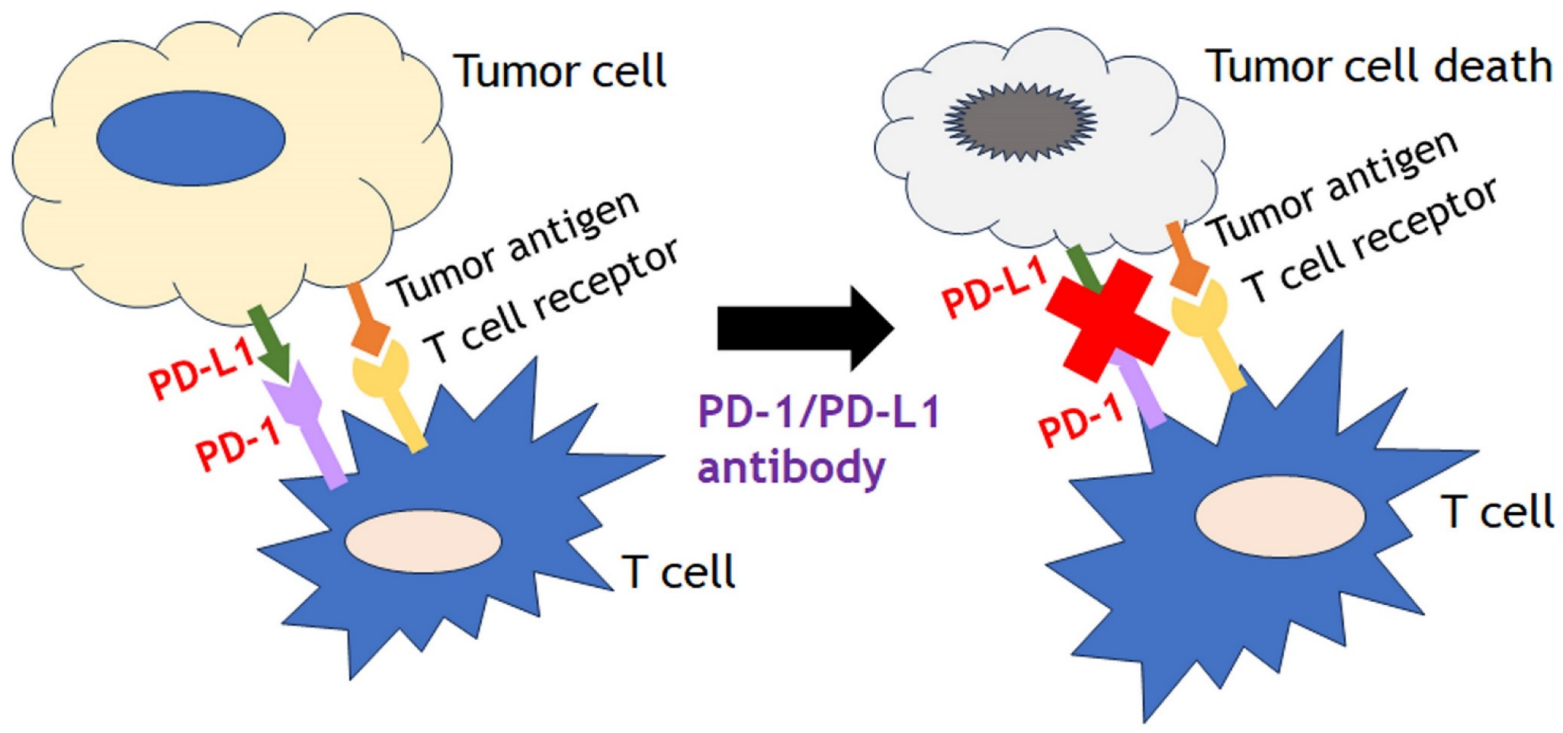
The binding of checkpoint protein PD-L1on cancer cells to PD-1on T cells keeps T cells from killing tumor cells in check. Blocking the binding of PD-L1 to PD-1 with an immune checkpoint inhibitor (anti-PD-L1 or anti-PD-1) allows the T cells to kill tumor cells.

Recent advances of immune-based cancer therapies can be divided three major categories such as cytokine-targeting therapies (interleukins, interferons), cell-based therapies (chimeric antigen receptor technologies) and immune checkpoint inhibitors (ICIs). Among them, one of the most growing modalities of immune modulatory therapies is ICIs, especially the programmed cell death receptor-1/ligand 1 (PD-1/L1) inhibitors. The binding of checkpoint protein PD-L1on cancer cells to PD-1on T cells keeps T cells from killing tumor cells in check. Blocking the binding of PD-L1 to PD-1 with an immune checkpoint inhibitor (anti-PD-L1 or anti-PD-1) allows the T cells to kill tumor cells (Fig. 6). They displayed promising efficacy in several solid tumor types as well as hematological malignancies. A meta-analysis of patients with pretreated microsatellite instability-high (MSI-H) cancers treated with ICIs found that ICIs were correlated with high activity, independent of tumor type and drug used 77. In this study, 939 patients across 14 studies were analyzed mainly in the pretreated settings, and the results showed that the pooled objective response rate was 41.5% (95% CI, 34.9%-48.4%), the pooled disease control rate was 62.8% (95% CI, 54.5%-70.3%), the pooled median progression free survival was 4.3 months (95% CI, 3.0-6.8 months), and the pooled median overall survival (OS) was 24 months (95% CI, 20.1-28.5 months). This study also showed that the pooled 1- and 2-year OS were 75.6% (95% CI, 61.8%-85.5%) and 56.5% (95% CI, 46%-66.4%), respectively. The positive outcomes after ICI treatment in MSI-H tumors with ICI could possibly be explained by the potential association with PD-L1 expression and the high mutation burden of those disease types. Concordantly, tumor mutational burden (TMB) is another emerged independent predictor of positive outcomes with ICIs treatment across various tumor types 78, 79. TMB is defined as the number of somatic mutations per DNA mega-base and originally measured using whole exome sequencing, but targeted NGS panel approaches as comprehensive genomic profiling (CGP) such as FoundationOne CDx assay (Foundation Medicine, Inc., Cambridge, MA) have taken place for a major role to estimate TMB recently. Those panels have been optimized to identify all types of molecular alterations (single nucleotide variants, insertion-deletion alterations, copy number alterations, and structural variants) in cancer related genes, as well as genomic signatures such as loss of heterozygosity, MSI and TMB in a single test.

Meanwhile, data related to the activity of ICIs in bone and soft tissue sarcomas are very scarce. A phase II study of PD-1 inhibitor pembrolizumab showed the ORR was 18% (7/40) in soft tissue sarcomas, 5% (1/22) in osteosarcomas and 20% (1/5) in chondrosarcomas 80. The pooled analysis of phase II clinical trials investigating anti-PD-1/L1 in patients with advanced soft tissue sarcomas has identified that the ORR was 15.1% and non-progression rate was 58.5% in 384 patients treated with anti-PD-1/L1 81. Among various histological types, patients with alveolar soft part sarcoma and undifferentiated pleomorphic sarcoma exhibited the highest response rates and leiomyosarcoma the lowest. Although ICIs might be effective in certain type of sarcomas, sarcomas mostly showed both MSI- and TMB-low, and low expression of PD-L1. In addition, sarcomas are considered to be so-called 'immune-oncologic cold' tumors which exhibit a scarce infiltration of immune cells in the tumors. Thus, the role of immune checkpoints is very limited in sarcomas and the stratification with those biomarkers for ICIs treatment in sarcomas are not much effective.

Cancer-related inflammation is one of the key players in cancer development and progression. Recently, several systemic inflammation-based scores including absolute lymphocyte count (ALC), neutrophil-to-lymphocyte ratio (NLR), platelet-to-lymphocyte ratio (PLR), lymphocyte-to-monocyte ratio (LMR), C-reactive protein (CRP), modified Glasgow prognostic score (GPS), and prognostic nutritional index (PNI), have been proposed as prognostic factors for several cancers 82, 83. In sarcomas, Liu et al reported that high levels of CRP (>10mg/L), GPS (>0), NLR (>2.57), PLR (>123.5), and low level of LMR (≤4.73) were significantly associated with adverse prognosis (P<0.05) in retrospective analysis of 162 osteosarcoma cases, and multivariate Cox regression analyses revealed that GPS, NLR, and occurrence of metastasis were top risk factors associated with death of osteosarcoma patients 84. Therefore, they proposed that these pre-treatment inflammation-based scores could be the independent prognostic factors for patients with osteosarcoma.

Eribulin is a novel microtuble-targeting chemotherapeutic agent which is a synthetic analogue of halichondrin B originally extracted from the marine sponge *Halichondria Okaida* 85. It was recently approved in many countries for the treatment of patients with unresectable or disseminated sarcomas, especially who have received a prior anthracycline-based regimen 86. The anticancer properties of eribulin are the suppression of microtuble growth and sequestration into non-functional aggregates through binding to a unique part of tubulin 87. Sato et al retrospectively analyzed inflammation-based scores in 53 patients who were treated with eribulin for recurrent or metastatic soft tissue sarcomas and found that pre-treatment NLR<3.0 was one of the independent predictive factors for durable clinical benefit and better progression-free survival. They concluded that baseline NLR could predicts the efficacy of eribulin for soft tissue sarcomas 88.

Although it has been reported that neutrophils interact with tumor cells by producing cytokines and chemokines, which affects the proliferation of tumor cells, angiogenesis and metastases 89 and lymphocytes play a major role in the immune response by mediating the immunologic destruction of various cancers 90, these inflammation-based indicators, especially NLR, have not been completely proved yet how they directly or indirectly affect the disease progression as well as response to the treatment. Upon the recent insights of inflammation-based indicators, further study will elucidate the details of the inflammation involvement in sarcoma progression.

Future perspectives: Basically, sarcomas are considered as so-called 'immune cold' tumors, such that a high bar still exists in front of us regarding the optimal use of immunotherapy against sarcomas. Several attempts including combination of PD-1 antibody and cytotoxic chemo agents or molecular targeting drugs have been conducted to make this therapeutic strategy more effective for this group of disease 91. Unfortunately, most attempts showed very limited effects, but investigation of combination strategies which convert 'cold tumors' into 'hot tumors' are on-going and hopefully would provide a novel insight in this field in the future.

## V. Tumor Microenvironment

Tumor microenvironment was added most lately in hallmarks of cancer. Like normal organ tissues, tumor tissue is composed of not only tumor cells, but also multiple stromal cell types and extracellular matrix proteins. These elements of tumor microenvironment are involved in structural and functional support for tumor growth. Cellular components of tumor environment include infiltrating stromal cells (cancer-associated fibroblasts; CAFs), immune cells (tumor-infiltrating lymphocytes: TILs, tumor-infiltrating macrophages: TAMs) and vascular-associate cells (endothelial cells; ECs, pericytes; PCs), and extracellular components include collagens, fibronectin, laminin and secreted growth factors and enzymes like matrix metalloproteinases.

**Figure 7:**
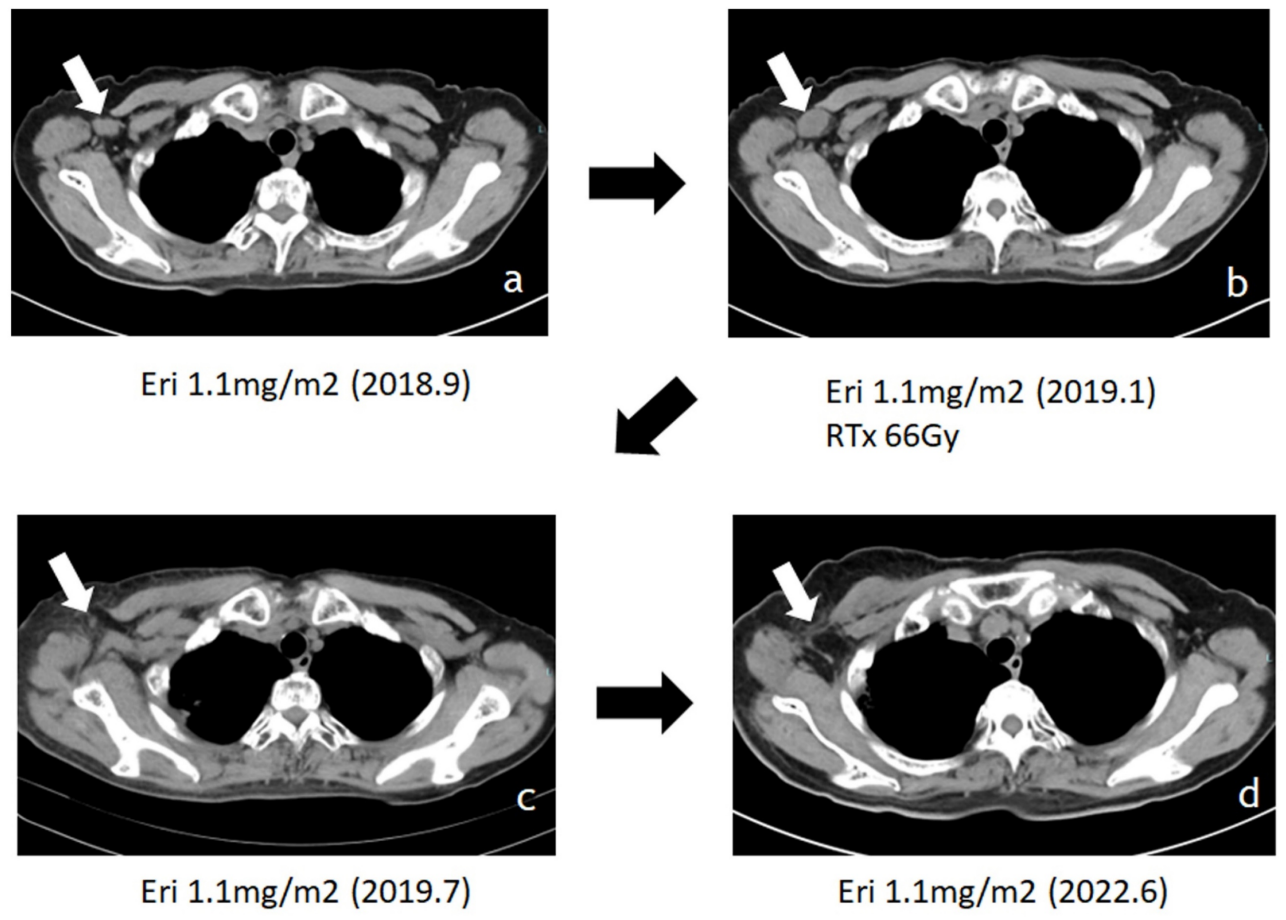
Additive effect of eribulin with irradiation has shown in advanced myxofibrosarcoma patient of our own case. Patient with axillary lymph node metastasis (LN mets, white arrow) who received concomitant administration of eribulin and irradiation onto LN mets showed the augmented effective with drastic shrinkage of tumor mass.: Treatment with eribulin alone allowed tumor enlargement after 4 months, then radiotherapy was added on eribulin (a to b), and tumor showed remarkable shrinkage after 6months of radiotherapy (b to c), then tumor shrinkage was maintained for more than 3 years with continuous eribulin treatment (c to d).

Attempt to target tumor microenvironment in sarcomas has been demonstrated in inhibition of angiogenesis 92 and immunomodulation 78 as a part of recent developments in molecular targeting treatments, and pazopanib which has the inhibitory effects against vascular endothelial growth factor receptor has been approved in many countries for advanced soft tissue sarcomas. Now, we will list eribulin on the table here again. Beside the well- known antitumor mechanism of eribulin, it suppresses clear cell sarcoma growth by inhibiting cell proliferation and inducing melanocytic differentiation both directly and via vascular remodeling 93. Other than sarcomas, eribulin promoted antitumor immune responses as well as mesenchymal-epithelial transition through the vascular remodeling in breast cancer 94, 95. Eribulin could reorganize tumor microvasculature which results in normalizing the microenvironment and inducing re-oxygenation in tumor tissues 96-98. This vascular reorganization decreased tumor hypoxia and carbonic anhydrase IX expression 94. Carbonic anhydrase IX (CA9) has been proposed as a marker for poor prognosis in soft tissue sarcoma 99 and inhibition of CA9 sensitizes to ionizing radiation in certain type of cancers 100. Vascular reorganization by eribulin in the tumors possibly contributes to sensitize the tumor cells to irradiation when radiation and eribulin were concomitantly given, which is evidenced by that radiation with eribulin significantly prolonged the survival of mice with intracerebral glioblastomas compared to that in mice treated with either radiation or eribulin alone 95. As a matter of fact, we have several experiences that patients with advanced sarcomas who received concomitant administration of eribulin and irradiation showed the augmented efficacy with drastic shrinkage of tumor mass (Fig. 7). Although we haven't examined the expression of CA9 and details of tumor vasculature reformation in those cases, it might be considerable that concomitant administration of eribulin, which modulates the tumor microenvironment, with irradiation could be effective treatment strategy for unresectable advanced sarcomas.

Regarding vascular remodeling and angiogenesis in tumor tissues, the cell traction force is a hallmark for endothelial cells that guide the formation of new blood vessels to supply oxygen and nutrients to the dormant cancer cells. Several mechanisms have been proposed to form the new vessels, and amongst of those, intussusceptive angiogenesis, a mechanism of a new blood vessel creation by splitting of an existing blood vessel in two, occurs in normal development as well as in pathologic conditions including tumor angiogenesis and vascular remodeling 98, 101. The other processes that could be involved in tumor angiogenesis are coalescent angiogenesis where capillaries fuse and form larger vessels to increase blood flow and circulation, and sprouting angiogenesis, vessel co-option and vessel elongation 102,103.

The interplay between cancer cells and immune cells during the above-mentioned cancer immunoediting process within the tumor microenvironment also creates unique immune environments, called 'tumor-immune microenvironment' (TIME). The TIME has been classified by several features including the infiltration patterns and inflammatory gene signatures of T cells, presence of B cells and tertiary lymphoid structures, PD-L1 expression and tumor mutational burden 104, 105. Although immune-based therapies against sarcomas have not significantly gained to date, but understanding immunoediting of the TIME through genomic instability and following neoantigen generation, T cell recognition and resultant tumor cell immunosuppressive programs could stratify tumors with different TIMEs that brings implications for cancer immunotherapy strategies in sarcomas in the future 106, 107. Recently, Stadtmauer et al reported a phase I clinical trial to evaluate the safety and feasibility of CRISPR-Cas9 gene editing in three patients with advanced cancer including myxoid/round cell liposarcoma 108. They conducted isolated T cells from the blood of a patient, and CRISPR-Cas9 gene editing were administered in those T cells to remove the TCR α chain gene (TRAC) and the TCR β chain gene (TRBC) and PDCD1 gene (encoding PD-1) loci. The cells were then transduced to express a TCR which was specific for the cancer-testis antigens NY-ESO-1, and these engineered T cells were returned to the patient by intravenous infusion. The results showed the modified T cells persisted for up to 9 months and patients were well tolerated, demonstrating the safety and feasibility and of CRISPR gene editing for cancer immunotherapy.

Finally, cancer cells are considered to undergo dormant state at G0-G1 cell cycle arrest and cellular reprogramming in the appropriate niche to adapt to survive. Targeting the interaction between cancer cell and their niche could inhibit the critical characteristics of cancers such as metastasis, however there are currently no effective means against sarcomas on this issue.

Future Perspectives: Directly targeting tumor microenvironment for sarcomas are still on the long way to go, however modulation of microenvironment such as tumor vasculature could potentially enhance the efficacy of other therapeutic means including chemo agents, radiotherapy and immunotherapies, such that optimal combination strategies should be investigated to improve the efficacy of various therapeutic modalities.

## VI. Tissue Invasion and Metastasis

**Figure 8:**
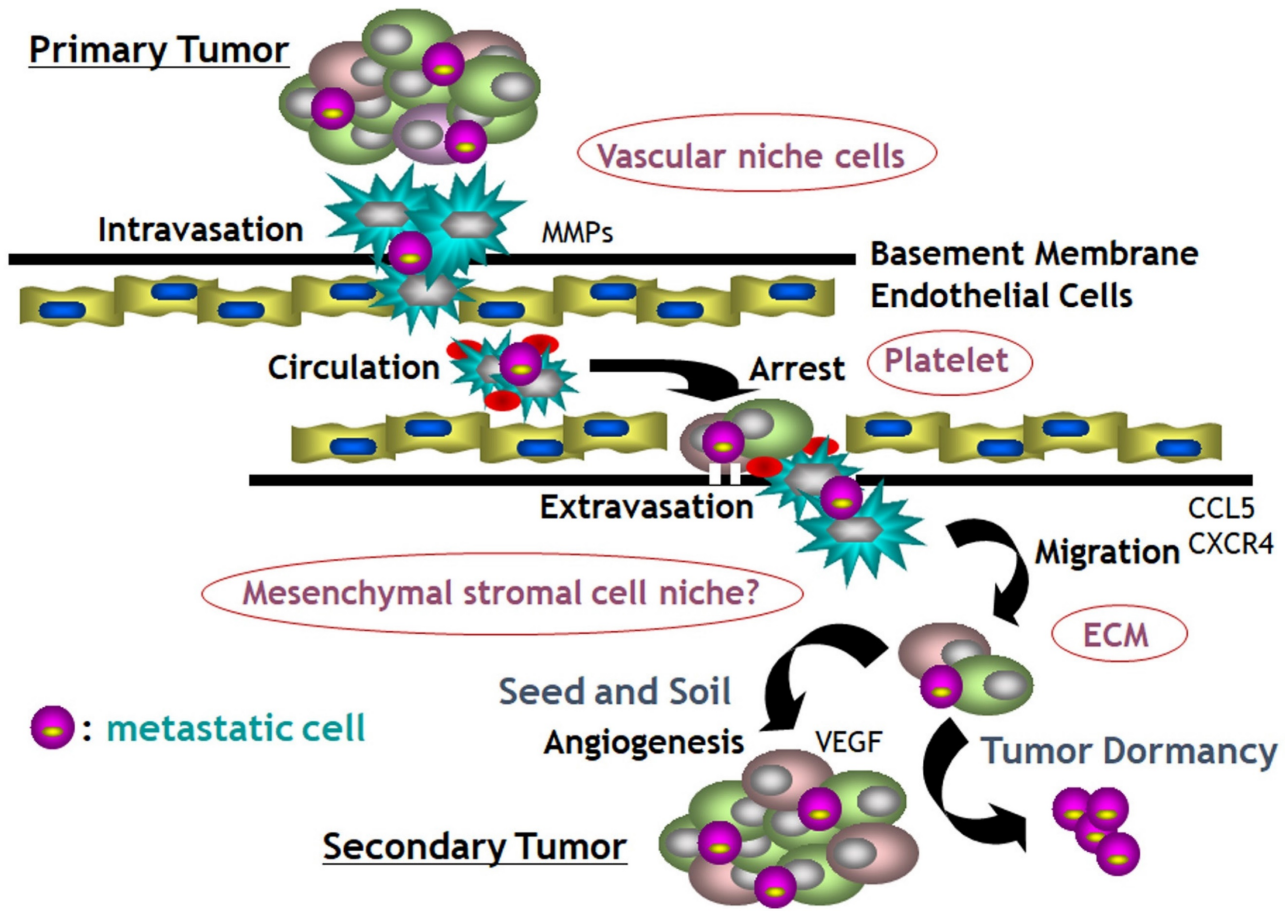
Growing evidence supports a collective route for invasion resulting in polyclonal metastasis and many collaborators like platelets, neutrophils and endothelial cells are involved in the metastatic cascade with numerous enzymes, growth factors and chemokines.

Invasion and metastasis are the feature defining the malignancy owing responsibility for over 90% of cancer-related deaths and metastasis involves a series of events as the 'invasion-metastasis cascade' 109. The 'invasion-metastasis cascade' is consisted of: invasion through the extracellular matrix such as basement membrane and stromal cells; intravasation into tumor vasculature; survival in the shear stress and immune attacks through circulation; extravasation at parenchyma of distant organs; survival and manipulation of foreign microenvironments; colonization and growth into metastatic foci with the formation of new vasculature 110. Classical metastatic cascade is based on the single cell metastasis depicted as the 'decathlon champ' model, however a hypothesis of collective route for invasion has emerged defining as polyclonal metastasis and many collaborators like platelets, neutrophils and endothelial cells are involved in the metastatic cascade (Fig. 8) 111, 112. Wrenn et al proposed that an intra-cluster heterogeneity during collective invasion would generate leader-follower dynamics within clusters 112. One or more “leader” cells at the front-most edge of the cluster could possibly be the stem-like cancer cells, and will direct the migration and remain connected to several “followers” behind along with the axis of migration as “front” and “rear” cells 112,113. These unique properties of tumor cell clusters that promote metastasis could provide promising potential targets for cancer treatments.

In this context, they proposed that blocking collective invasion could be the potential strategies against metastasis by targeting leader cell activity, circulating tumor cell (CTC) clusters in the circulation, enhancing the immune system to diminish disseminated clusters and micro-metastases to prevent their expansion 114-118. In terms of diagnostic values, detecting CTCs and circulating tumor DNAs (ctDNAs) are on the way by liquid biopsy or NGS using blood samples from cancer patients such as FoundationOne liquid CDx (Foundation Medicine, Inc. Cambridge, MA) and Guardant360® CDx (Guardant Health Japan Corp, Tokyo).

In addition to targeting the individual metastatic cells, targeting the collaboration amongst cells that collectively promotes their metastatic potential such as critical secreted paracrine loops, juxtracrine interactions, and the structures which facilitate intercellular communication could be another strategy against metastasis. A recent study found that interrupting integrin signaling generated by collectively invading sarcoma cells could enhance the efficacy of radiotherapy 119, and disrupting cell-cell communication may interrupt the acquisition of highly proliferative, aggressive phenotypes in cancer cells 120.

Numerous reports have suggested that platelets play a role in facilitating metastasis by protecting circulating tumor cells from the shear stress of the bloodstream, from immunological assault during their intravascular phase and by mediating tumor cell adhesion and embolization in the microvasculature of secondary organs 121. Platelets also enhance the proliferation and motility of tumor and endothelial cells promoting tumor growth, metastasis, and angiogenesis by releasing bioactive molecules 122, including peptide mediators such as platelet-derived growth factor (PDGF), vascular endothelial growth factor (VEGF), transforming growth factor-β (TGF-β), chemokine ligands (CXCL) 4,7 and 12 123,124 as well as lipid mediators such as thromboxane A2 (TxA2), sphingosine-1-phosphate (S1P) and lysophosphatidic acid (LPA) 125,126. Takagi et al identified that osteosarcoma cells commonly exhibit high platelet activation-inducing characteristics, and molecules released from activated platelets promote the invasiveness of osteosarcoma cells. They indicated that osteosarcoma-induced platelet activation leads to abundant release of molecules including LPA and exposure to those releasates induces morphological changes and increase the invasiveness of osteosarcoma cells. They found that LPA receptor 1 (LPAR1) is notably upregulated in osteosarcoma, such that the pharmacological inhibition of LPAR1 by the orally available LPAR1 antagonist, ONO-7300243, prevented pulmonary metastasis of osteosarcoma in the mouse models, indicating that the LPA-LPAR1 axis is essential for the osteosarcoma invasion and metastasis, and targeting LPAR1 would be a promising therapeutic intervention for advanced osteosarcoma 127.

Since metastasis promoting intercellular cooperation involves throughout the entire metastatic cascade, multiple anti-collective strategy should be developed throughout the metastatic process of invasion, circulation, and colonization phases. Although it remains uncovered whether such strategies could be adapted to destroy collectively metastasizing cancers, more critical inter-cellular interactions could be identified in cancer cell collectives eventually, and hope some of those may turn out to be fruitful clinical targets.

Future Perspectives: We do not have any specific means to prevent and treat metastasis in sarcomas. As forementioned, mitochondria are transferred from the platelets to cancer cells and reprogram the metastatic state of cancer cells 72, and this is just an example to indicate that the interaction with cancer cells and stromal cells like platelets, fibroblasts and immune cells could be a potential target in cancer cell metastasis. Further investigation of these mechanisms could contribute to overcome the life-threatening metastatic disease.

## Conclusions and Future Perspectives

**Figure 9:**
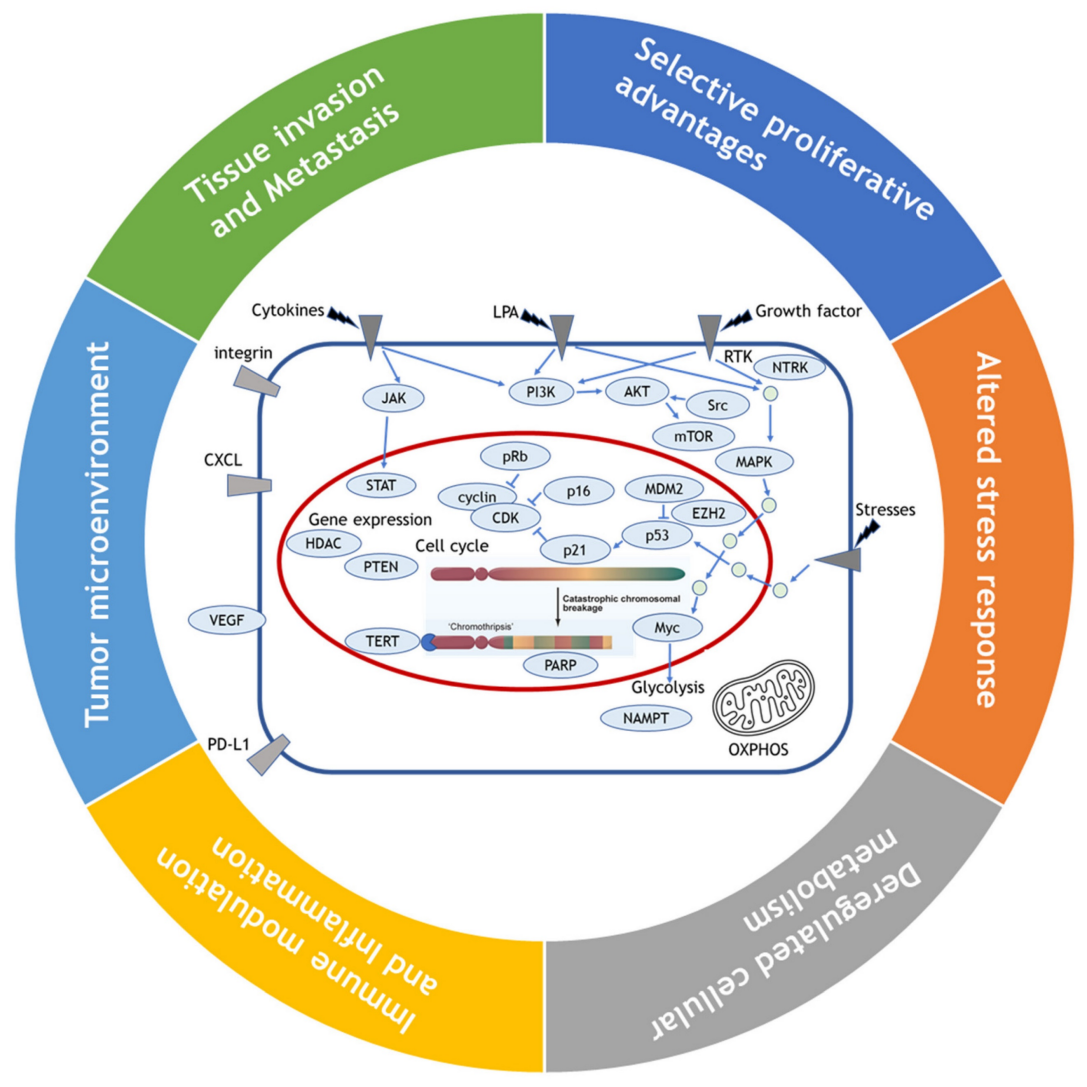
The summary of reorganized hallmarks and possible targetable factors are depicted. Number of targets are involved in signaling pathways downstream of growth factors, cytokines as well as stress responses.

Thanks for the concept of hallmarks framework, cancer research has once been considered to progress straightforward. Responsible genetic changes for these hallmarks could establish the molecular mechanisms of cancer, and could take a step toward the application of those mechanisms for both diagnostic and treatment regimens. However, the reality of cancer biology is far more complicated, and number of attempts based on these hallmarks have been insufficient in clinical settings. As a matter of fact, there are so many genetic or epigenetic mutations/alterations found year by year or even day by day, so that the cancer genetic landscape is becoming highly diverse more and more and there is no one-to-one linkage between genetic changes and hallmarks. Thus, we thought that re-organizing the concept of the cancer hallmark framework would be needed, and we depicted the schematic summary of reorganized hallmarks and possible targetable factors in each hallmark, indicating the crossover of most targets in current treatment strategies densely related to the downstream pathways of growth factor receptors and stress responses (Fig. 9).

Along with the expansion of the number of newly identified genetic alterations, the list of hallmarks should be expanded to establish the linkage between the hallmarks and genetic alterations. On the other hand, there is a high degree of overlap and dynamics for these hallmarks, i.e., the same gene alteration can be involved in different hallmarks, and the different gene alterations can be involved in the same hallmark, and another example is a hallmark might require additional hallmarks, i.e., invasion and metastasis incorporate many other hallmarks such as proliferative advantages, altered metabolism and tumor microenvironment. Therefore, different hallmarks could be unified into a certain alternative hallmark, for instance sustained growth signal and evading anti-growth signal into selective proliferative advantages. We cannot tell which hallmarks are more important than others and which hallmarks we target first. Recent advances of sarcoma treatments have been limited to targeting the hallmark of proliferative advantages such as tyrosine kinase growth factor receptors inhibitors. Furthermore, there is an aspect of cancer cell characteristics like cytoskeletons including actin, myosin, and microtubules, which are undefined in a cancer hallmark such as cell confinement, could be an emerging field of cancer therapy. For instance, eribulin which inhibits microtubule polymerizations is approved for soft tissue sarcomas.

The altered stress response represented by genome instability would possibly be the foremost hallmark, because the significant stress could result in increased system dynamics, generating genome level heterogeneity related to the tumor heterogeneity, which would be a fundamental mechanism of oncogenesis and hardly targetable. However, it seems many of us put every hallmark more or less equally important. Identification of the novel framework which can be linked to any of the individual hallmarks possibly lead the future direction for adapting the hallmarks to the selection of the targets in each patient.

We have summarized the topics raised here regarding hallmarks and their targets, available treatments, and future perspectives in sarcomas in Table 1, however we cannot cover every aspect of cancer hallmarks and implications of all hallmarks for clinical settings. Understanding the molecular basis of these hallmarks are still on the way to fulfill the lack of knowledge of cancer biology as well as their clinical relevance. The gap between the conceptual understanding and clinical applicability of the biological principles and molecular mechanisms will be filled in based on the further knowledge of genomic alteration obtained from comprehensive high-resolution analysis such as NGS in conjunction with the hallmarks of cancer.

On the other hand, Gyawali et al claimed that the cancer field seems losing the sight of what matters to patients, despite the understanding of the molecular details in cancer biology, important progress in some elements of cancer care, and draggable targets showing spectacular improvements 128. They raised a caution that clinical benefits have been limited with numerous newly approved cancer therapeutics so far, yet they are still prescribed to patients, and unfortunately this is not exceptional in sarcoma patients, claiming that oncology needs a 'common-sense revolution' and should prioritize treatments that effectively improve both survival and quality of life. Cancer research has been conducted heavily on 'Moon shot' Initiatives in decades. Yes, there is no doubt that the molecular mechanisms are very much important, but it does not imply everything can be targeted, and we need a bit more restrictive with developing new therapies. On this view, we may also think about a re-calibration for supporting low-tech inexpensive 'Ground Shot' interventions that could improve outcomes for many patients especially with sarcomas 129, 130.

In our knowledge, this is the first attempt to overview and reshape the current comprehension of 'hallmarks of cancer' in sarcomas and hope this could be helpful to think about what sarcomas are and how we should face to them. We have tried to include as much up-to-date information as possible, but most of them are the rapidly evolving area time-to-time, thus we also set a goal for this paper as every reader being able to add the latest up-to-date understandings by themselves.

## Figures and Tables

**Table 1:** Cancer hallmarks and examples of current treatments and their biomarkers, and possible future perspectives for each hallmark

Hallmark in Cancer/Sarcoma	Possible Targets	Current Treatments	Possible Biomarkers	Future Perspectives
Selective Proliferative Advantages	receptor tyrosine kinase	tyrosine kinase inhibitor (imatinib, sunitinib, pazopanib,larotrectinib, entrectinib)	growth factor expression	combinatory treatments with either conventional chemothrapeutic agnets or molecular targeting agents
	MAPK, PI3K/AKT/mTOR, JAK/STAT	IGF1R inhibitor, mTOR inhibitor		
	MET, Src	MET inhibitor (crizotinib) Src inhibitor (dasatinib)	MET, Src expression	
	cell cycle regulators	CDK4/6 inhibitor (palvociclib, ribociclib)	endogeneous CDK4, CCND, CCNE, RB1, E2F1, and CDKN2A alteration	HDAC inhibitor, MDM2 inhibitor stratification
	epigenetic regualtion	EZH2 inhibitor (tazemetstat)	SMARCB1 (BAF47INI1) deltion	epigenetic regulators
Altered Stress Response (genetic instability)	chromosomal alteration (chromothripsis, chromplexy)	N/A	TERT activation fusion gene	TERT inhibitor
				targeting fusion genes
Deregulated Cellular Metabolism	PI3K/Akt/mTOR	mTOR inhibitors (rapamycin, temsilolimus, everolimus)	growth factor expression Src expression	combinatory treatments
	Src	Src inhibitor (dasatinib)		
	Poly (ADP-ribose) polymerases (PARP)	PARP inhibitors	specific DNA-repair defects	combinatory treatments of PARPis and NAMPTis
	nicotinamide phosphoribosyl transferase (NAMPT)	NAMPT inhibitors	NAMPT expression	
	mitochondria OXPHOS	N/A	N/A	phytochemical stilbenes
Immune Modulation and Inflammation	immune checkpoint	PD-1/L1 inhibitors (pembrolizumab) CTLA-4 inhibitors	microsatellite instability (MSI) tumor mutaion burden (TMB) PD-L1 expression	stratification combinatory treatment
	cytokines	N/A	interleukin or interferon expression	interleukin or interferon modulators
	microtubles	eribulin	neutrophil lymphocyte ratio (NLR)	stratification
Tumor Microenvironment	angiogenesis	tyrosine kinase inhibitor (pazopanib, eribulin)	VEGF, CA9 expression,	combinatory treatment with irradiation
	tumor immune environment	N/A	T cell infiltration patterns T cell inflammatory gene signatures	gene editing T cells
	T cell receptor (TCR), PD-1	N/A	N/A	gene editing T cells
Tissue Invasion and Metastasis	circulating tumor cells (CTCs)	conventional chemotherapy	circulation tumor DNA (ctDNA)	liquid biopy (diagnostic)* ex vivo* activated NK cells
	cell-cell communications	N/A	integrin expression	integrin interruption multiple anti-collective therapies
	secreted paracrine molecules	N/A	PDGF, VEGF, TGF-β, CXCL, TxA2, S1P, LPA	LPAR1 antagonist

MAPK: mitogen-activated protein kinase; PI3K: phosphatidylinositol-3 kinase; mTOR: mammalian target of rapamycin; JAK: Janus kinase; STAT: signal transducers and activators of transcription; IGF1R: insulin-like growth factor 1 receptor; CTLA-4: cytotoxic T-lymphocyte-associated antigen; VEGF: vascular endothelial growth factor; CA9: carbonic anhydrase 9; PDGF: platelet-derived growth factor: TGF-β: transforming growth factor-beta: CSCL: chemokine (C-X-C motif) ligand; TxA2: thromboxane A2; S1P: sphingosine-1-phosphate; LPA: lysophosphatidic acid.
